# Impact of magnesium sulfate therapy in improvement of renal functions in high fat diet-induced diabetic rats and their offspring

**DOI:** 10.1038/s41598-023-29540-w

**Published:** 2023-02-08

**Authors:** Mohammad Vahid Touliat, Hossein Rezazadeh, Mehran Beyki, Sajad Maghareh-Dehkordi, Mohammadreza Sharifi, Ardeshir Talebi, Nepton Soltani

**Affiliations:** 1 Department of Biotechnology, Pharmacy School, Isfahan University of Medical Sciences, Isfahan, Iran; 2 Department of Physiology, School of Medicine, Isfahan University of Medical Sciences, Isfahan, Iran; 3 Department of Genetics and Molecular Biology, School of Medicine, Isfahan University of Medical Sciences, Isfahan, Iran; 4 Department of Clinical Pathology, School of Medicine, Isfahan University of Medical Sciences, Isfahan, Iran

**Keywords:** Endocrinology, Medical research

## Abstract

The role of magnesium sulfate (MgSO_4_) administration to prevent diabetic nephropathy (DN) by reducing insulin resistance (IR) and the relationship of this action with gender and the expression of NOX4 and ICAM1 genes in the parents and their offspring were studied. Males and females rat, and their pups were used. Type 2 diabetes induced by high-fat diet (HFD) administration and a low dose of streptozotocin. Animals were divided into the: non-treated diabetic (DC), the diabetic group received insulin (Ins), and the diabetic group received MgSO_4_. Two groups of parents received just a normal diet (NDC). Following each set of parents for 16 weeks and their pups for 4 months, while eating normally. We assessed the amount of water consumed, urine volume, and blood glucose level. The levels of glucose, albumin, and creatinine in the urine were also measured, as well as the amounts of sodium, albumin, and creatinine in the serum. Calculations were made for glomerular filtration rate (GFR) and the excretion rates of Na and glucose fractions (FE Na and FE G, respectively). The hyperinsulinemic-euglycemic clamp was done. *NOX4* and *ICAM1* gene expressions in the kidney were also measured. MgSO_4_ or insulin therapy decreased blood glucose, IR, and improved GFR, FE Na, and FE G in both parents and their offspring compared to D group. MgSO_4_ improved *NOX4* and *ICAM1* gene expressions in the parents and their offspring compared to D group. Our results indicated that MgSO_4_ could reduce blood glucose levels and insulin resistance, and it could improve kidney function.

## Introduction

Diabetes mellitus (DM) is a metabolic disorder closely associated with significant and chronic microvascular and macrovascular complications spreading worldwide^1,2^. One of the most life-threatening complications which were gone along with is diabetic nephropathy (DN). Initially firmly attributed to DM, DN may be brought on by a gradual decline in glomerular filtration rate (GFR), arterial hypertension, and an increase in cardiovascular morbidity and mortality^3,4^. DN can be defined as a spectrum of distinctive structural and functional changes, including glomerular hyperfiltration in the very early disease stage and the presence of moderately increased albuminuria which is called microalbuminuria frequently seen in the patients with DM^5,6^. To effectively cure DN development, metabolic and hemodynamic abnormalities must be well controlled. Keeping proper glucose under control has reportedly been shown to reduce diabetic microvascular issues^7^. This suggests that patients with type 2 diabetes who received rigorous glucose therapy had a lower risk of developing renal failure^8^. NOX4 (NADPH oxidase 4) and ICAM1 (intercellular adhesion molecule 1) are two important genes whose expressions were considered basic characteristics kidneys signaling pathways^9,10^. NOX4 is located in mitochondria and plasma membrane, and its increased expression has a role in renal damage by enhancing podocyte apoptosis and mesangial cell proliferation^11^. Many studies showed that NOX4 has a crucial role to produce the superoxide ions and hydrogen peroxide in DN^12,13^, and it participates in cardiovascular disorders^14^. The important note is that NOX4 was closely linked to increased ROS production when its overexpression in mRNA and protein results in renal injuries^15,16^.

ICAM1 is a cell surface glycoprotein which expressed in endothelial cells and leukocytes in the immune system^17,18^. According to reports, this gene may be very important in the pathogenesis of DM and DN^12,13^. ICAM1 is substantially linked with albuminuria in type 2 diabetes (T2D) patients, both at the mRNA and protein levels^19^. According to animal trials, ICAM1 levels in streptozotocin (STZ)-induced diabetic rats increased, respectively, compared to non-diabetic ones, and furthermore, its overexpression could be seen in glomeruli diabetic rats^20^.

Some studies indicated that low blood concentration of magnesium (Mg) is closely associated with DM^21,22^. Mg deficiency was linked with other important complications, such as a macrovascular and microvascular disease like DN^23^. There is evidence that, despite adequate and acceptable amounts of magnesium being present in the serum, insulin resistance (IR) brought on by diabetes mellitus (DM) may significantly reduce the amount of magnesium that enters cells and worsen cell function^24,25^. According to our previous findings, magnesium sulfate (MgSO_4_) therapy can improve insulin sensitivity by increasing GLUT4 (glucose transporter number 4) gene expression and furthermore^26,27^, MgSO_4_ administration in STZ-induced diabetic rat model could improve renal function^28^.

The current research aimed to answer two main questions: (1) whether DN in T2D rats may be prevented by MgSO_4_ injection via lowering IR, and if this effect varies according to gender. (2) Examine the expression of NOX4 and ICAM1 in offspring of T2D male and female animals to determine the connection between the administration of MgSO_4_ and the prevention of DN. (3) Identify the parameters of renal kidney functioning and MgSO_4_'s activity to lower blood glucose.

## Materials and methods

### Ethical statement

All experiment protocols were performed based on relevant guidelines and regulations. All animal experiments were performed based on ARRIVE (Animal Research: Reporting of in Vivo Experiments) guidelines and approved in advance by Ethical Committee of Isfahan University of Medical Sciences (Ethics code # IR.MUI.RESEARCH.REC.1398.535). All methods are reported based on ARRIVE guidelines.

#### Animals

Forty-eight males and females Wistar rats (4 weeks old, weighing 80–90 g) and their offspring were used. The animals were housed at standard condition (constant temperature of 22 ± 2 °C and 12 h light/12 h dark cycle and free access to water and rat chow).

### Type 2 diabetes induction

For type 2 diabetes induction, thirty-six animals received a high-fat diet (HFD) including 58% fat, 25% protein, and 17% carbohydrate (Table 1)^29^ for 3 months, and then they received 35 mg/kg of STZ (Sigma Aldrich, Hamburg, Germany) via intraperitoneal IP injection^30,31^.

**Table 1:** Components of high fat diet.

Components	g/kg
Powdered NPD	365
Lard	310
Casein	250
Cholesterol	10
Vitamin and mineral mix	60
DL-Methionine	3
Yeast powder	1
Sodium chloride	1

### Experimental design

**Figure 1:**
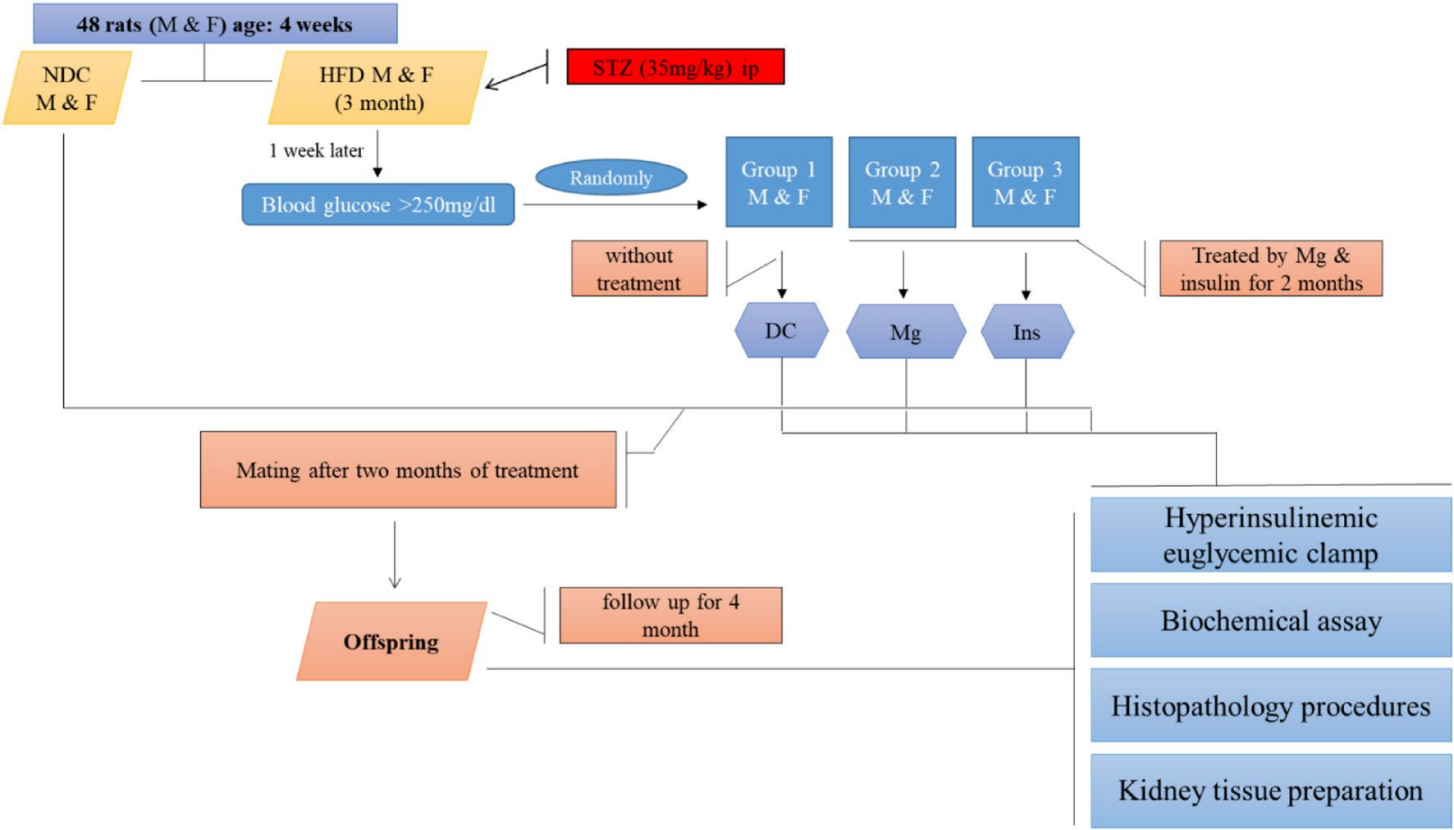
Experimental design, 48 male (M) and female (F) rats at 4 weeks old, weighing 80–90 g are used in the study.

Animals with blood glucose levels over 250 mg/dl and impaired intraperitoneal glucose tolerance test (IPGTT) results were included to confirm the presence of type 2 diabetes one week after STZ administration. The included animals were randomly divided into three groups (N = 6) under the name non-treated diabetic (D), the diabetic group treated with insulin 2.5 U/kg twice a day (Ins), and the diabetic group treated with 10 g/l MgSO_4_ (Sigma Aldrich, Hamburg, Germany) via drinking water (Mg)^27,32^. HFD was continued for all diabetic groups until the end of the study. One group in each sex (N = 6) was kept as a non-diabetic control group (NDC), and they just received a normal diet. All animals were followed for 16 weeks, and the blood glucose level and body weight were determined every month (Fig. 1) (Table 2).

**Table 2:** Summery of the resells.

	P		O		P		O		P		O		P		O	
	M	F	M	F	M	F	M	F	M	F	M	F	M	F	M	F
GIR	N	N	N	N	↓	↓	↓	↓	↑	↑	↑	↑	↑	↑	↑	↑
BG	N	N	N	N	↑	↑	–	–	↓	↓	–	–	↓	↓	–	–
WC	N	N	N	N	↑	↑	–	–	↓	↓	–	–	↓	↓	–	↓
UV	N	N	N	N	↑	↑	–	–	↓	↓	–	–	↓	↓	–	–
GFR	N	N	N	N	↑	↑	↑	↑	↓	↓	–	↓	↓	↓	–	–
FE Na	N	N	N	N	↓	↓	↓	–	–	↑	–	–	↑	↑	–	–
FE G	N	N	N	N	↓	↓	–	–	↑	↑	↑	↓	↑	↑	↑	↓
UG	N	N	N	N	↑	↑	–	↑	↓	↓	–	↓	↓	↓	–	↓
UA	N	N	N	N	↑	↑	↑	↑	↓	↓	–	–	↓	↓	–	–
UU	N	N	N	N	↑	↑	↑	↑	–	–	–	–	–	–	–	–
KW	N	N	N	N	↑	↑	↑	–	↓	↓	↑	↑	–	↓	↑	↑
NOX4	N	N	N	N	↑	↑	↑	↑	↓	–	–	↓	↓	↓	–	–
ICAM1	N	N	N	N	↑	–	–	↑	↓	–	↓	↓	↓	–	↓	↓

↑: Increase, ↑: Decrease, –: No change, N: normal.

*P* Parents, *O* Offspring, *GIR* Glucose infusion rate, *BG* Blood glucose, *WC* Water consumption, *UV* Urine volume, *FE Na* Sodium fraction excretion rate, *FE G* Glucose fraction excretion rate, *UG* Urine glucose, *UA* Urinary albumin, *UU* Urinary urea, *KW* Kidney weight.

The metabolic cage measured urine volume and water consumption every month. To perform this test, each rat was put in a metabolic cage (Tajhizgostar Co, Iran) for 24 h.

Males and females from each group were separated and let to breed after eight weeks of therapy.

(Each male could couple with only one female to prevent genetic admixture). After giving birth, the mother animals nursed their young for a month while keeping the male animals apart. Every animal received HFD throughout mating, pregnancy, and lactation. The number of male and female offspring born in multiple groups was different. However, among the offspring born in each group, 6 males and 6 females.

### Surgery

A single dose of ketamine (100 mg/kg) and xylazine (8 mg/kg) was used to anesthetize the animals and common carotid artery and jugular vein were cannulated by heparinized polyethylene 50 tubes. Finally, the tubes were moved to the back of animal and fixed there. The animals go back to their cage for recovery. During 3–5 days’ recovery, 150 U/ml of heparinized salines was injected into both tubes on a daily basis.

### Euglycemic-hyperinsulinemic clamp in the conscious rat

After 3–5 days of recovery, the amounts of insulin and glucose infusion rate were determined based on the weight of all animals after 17 h of fasting^33^. The jugular vein was connected to two microinjection pumps (New Era Pump System Inc. Farmingdale, NY, USA) to inject insulin and glucose simultaneously via a Y connector, and the carotid artery was used for blood sampling. Based on weight, 20 mU/kg/min insulin was administrated at a rate of 5 ml/h, and 25% of the glucose was injected with a variable rate to clamp glucose at euglycemia. The clamp was left on each animal for five hours. A glucometer (Ascensia ELITE XL glucometer) was used to measure blood sugar levels every 5–10 min, and 10U/ml heparinized saline was replaced. The quantity of glucose administered during the final 30 min of the clamp was used to calculate insulin-stimulated glucose uptake. It was used to calculate the amount of glucose infusion rate (GIR: mg/kg/min).

### Biochemical assay

Blood urea nitrogen (BUN) and serum creatinine (Cr) were measured by Olympus Medical Instruments (lot N BUN: biorex 126-01-057, lot N Cr: biorex 11701049). Serum sodium (Na) levels were determined using Caretium Medical Instruments (lot N: Ae-04180, 09031). Urine glucose, Cr, and albumin levels were measured using Olympus Medical Instruments (lot N BUN: biorex 126-01-057, lot N Cr: biorex 11701049). GFR was calculated from plasma Cr clearance, and sodium fraction excretion rate (FE Na) and glucose fraction excretion rate (FE G) were also calculated.

### Histopathology procedures

Paraffin-embedded tissues were used for histopathological staining. The periodic acid Schiff staining (PAS) and jones methenamine silver (JMS) were used to examine the tissue injury. To consider the kidney damage, the presence of tubular atrophy, fibrosis, connective tissue changes, inflammation, degeneration of tubular epithelial, congestion, glomerular damage were evaluated. Based on damage intensity, the samples were scored as 1–4, while score zero was considered normal tissue.

### Kidney tissue preparation and Real time- PCR process

Twenty-four hours after hyperinsulinemic-euglycemic clamp was done, the animals were anesthetized with ketamine (100 mg/kg) and xylazine (8 mg/kg), and blood was taken from the heart, then the left kidney was removed, washed in ice cold isotonic saline. Afterward, 50–100 mg of tissue was kept in DNase/RNase free microtube and immediately put in a nitrogen tank. It was then stored at − 80 °C, ahead of RNA isolation.

Based on manufacturer's guidelines, five to ten ng of the total RNA was applied for synthesizing complementary DNAs (cDNAs) using M-MLV RT (Anacell, lot N: CS0021). To calculate mRNA expression level, quantitative real-time PCR (qPCR) was performed with SYBR-green at step one real-time PCR machine (Applied Biosystems). One microliter of the total cDNA was mixed with 10 µl of 2 × SYBR Green PCR mix with ROX, treated with sterile water, and 10 pmol/ml of each sense and antisense primers for the measured genes. Table 3 shows the primers used with an initial denaturation temperature of 95 °C. The input cDNA was normalized using beta-average actin's expression as an internal reference gene. The relative expression values were determined using comparative CT approach^34^.

**Table 3:** Primers for quantitative real-time PCR analysis of gene expression.

Gene	Reveres primer	Forward primer	Reference
Beta-actin BLAST	CTGACCCATACCCACCATCAC	ACAACCTTCTTGCAGCTCCTC	Designed with NCBI’s Primer
NOX4 BLAST	CCTGCTAGGGACCTTCTGTG	TGGGCCTAGGATTGTGTTTGA	Designed with NCBI’s Primer
ICAM1	CGCTCTGGGAACGAATACACA	AAGCTCTTCAAGCTGAGCGA	Designed with NCBI's Primer BLAST

### Ethical approval

This study protocol was approved by the Ethics Committee of Isfahan University of Medical Science in Iran.

## Results

**Figure 2:**
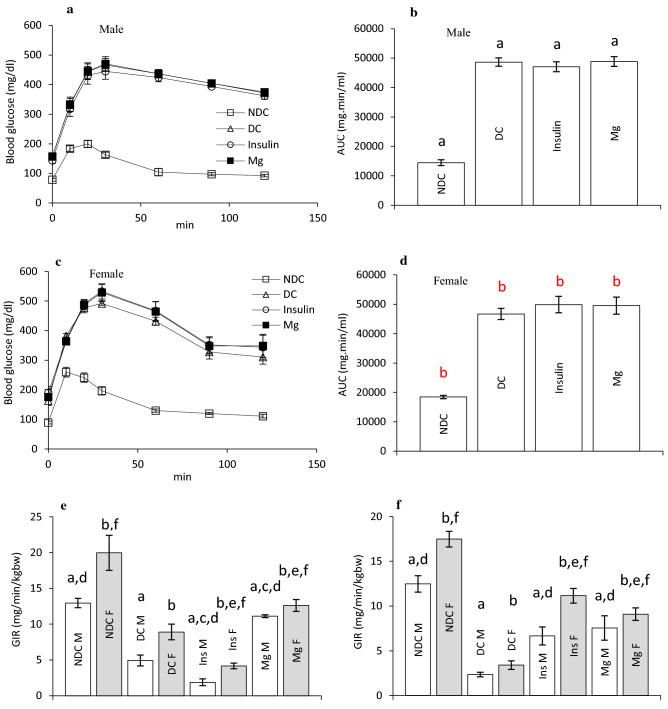
Comparison of intraperitoneal glucose tolerance test (IPGTT) in male (**a**) and female (**c**) before intervention, the area under the glycemic curve (AUC) (**b** in male and d in female), glucose infusion rate (GIR) in parents (**e**) and their offspring (**f**) in non-diabetic control group (NDC) was fed with normal diet, to preform IPGTT, the animals were fasted overnight for 15 h and in the day after, the animals received 1.5 g/kg glucose via IP injection. And the blood glucose was measured by using of glucometer (Ascensia ELITE XL glucometer) through a tail vein at 0, 20, 30, 40, 60, 90, and 120 min after glucose administration. diabetic group received high-fat diet and 35 mg/Kg STZ (D), diabetic animals treated with insulin (2.5 U/Kg two time per day) (Ins) and diabetic animals treated with 10 g/l magnesium sulfate via drinking water (Mg). 10 rats in each group and data are expressed as mean ± SEM. a Significant difference between D male group and other male groups (*P* < 0.0001). (**b**) Significant difference between D female group and other female group (*P* < 0.001). (**c**) Significant difference between male Mg group and male insulin group (*P* < 0.001). (**d**) Significant difference between male NDC and male Mg and male insulin groups (*P* < 0.001). (**e**) Significant difference between female Mg group and female insulin group (*P* < 0.001). (**f**) Significant difference between female NDC and female Mg and female insulin groups (*P* < 0.001).

IPGTT was preformed after diabetes induction in our groups to confirm the T2D induction. Our results showed that in all diabetic animal’s blood glucose increased after glucose injection and it could not reach to the normal values 2 h after glucose administration in both sexes and the area under the curve (AUC) of glycemic curve in both sexes is higher than non-diabetic control (NDC) group (male: *p* < 0.0001, female: *p* < 0.001, Fig. 2a–d).

Our results also showed that diabetes induction significantly decreased GIR in both parents in compared to both male and female NDC groups (male: *p* < 0.0001, female: *p* < 0.001). Administration of insulin or MgSO_4_, significantly increased GIR in male (*p* < 0.001) and female (*p* < 0.001) of treated groups compare to male and female of DC group (Fig. 2e). The findings of this study also showed that GIR significantly (male: *p* < 0.0001, female: *p* < 0.001) decreased in both sexes’ offspring of diabetic parents 4 months after birth in compared to the both sexes of NDC parents (Fig. 2f). And this data was significantly (*p* < 0.001) rises in both sex’s offspring of Mg-treated and insulin-treated parents in compared to male and female offspring of DC groups (Fig. 2f).

**Figure 3:**
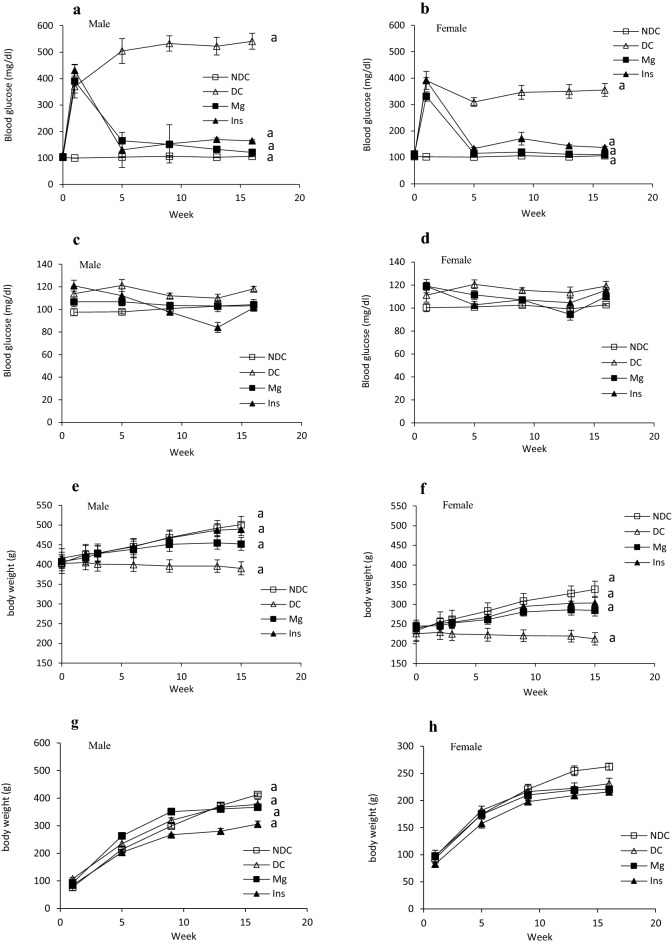
Comparison of fed blood glucose in male and female parents and their male and female offspring (**a**, **b**, **c** and **d** respectively) and body weight in male and female parents and their male and female offspring (**e**, **f**, **g** and **h** respectively) in non-diabetic control group (NDC) was fed with normal diet, diabetic group received high-fat diet and 35 mg/Kg STZ (D), diabetic animals treated with insulin (2.5 U/Kg two time per day) (Ins) and diabetic animals treated with 10 g/l magnesium sulfate via drinking water (Mg). 10 rats in each group and data are expressed as mean ± SEM. (**a**) Significant difference between D male and female groups and other male and female groups (*P* < 0.0001).

Diabetes induction increased the blood glucose level from 106 ± 2.2 to 541 ± 29.9 mg/dl in male groups and from 106 ± 2.4 to 355.4 ± 24.3 mg/dl in female groups. Administration of MgSO_4_ or insulin in both diabetic parents for 16 weeks significantly (*p* < 0.0001) decreased blood glucose level in compared to DC groups (Fig. 3a and b). We have not observed a significant difference between the blood glucose level in all offspring groups (Fig. 3c and d). Body weight could not increase in non-treated diabetic groups (from 402.5 ± 18.6 to 390.38 ± 16.4 g in male group and from 225.75 ± 17.1 to 213.3 ± 15.5 g in female group), but MgSO_4_ (from 407.3 ± 17.7 to 452.2 ± 16.04 g in Mg-treated male group and from 245 ± 10.2 to 284.7 ± 14.2 g in Mg-treated female group) or insulin (from 416 ± 24.3 to 489.4 ± 16.3 g in insulin-treated male group and from 238 ± 12.01 to 303.6 ± 16.5 g in insulin-treated female group) therapy could significantly (*p* < 0.0001) increase the body weight in both parents after 16 weeks administration in compared to DC animals (Fig. 3e and f). We did not observe any significant differences between body weight in all males and female’s offspring of all parents (Fig. 3g and h).

**Figure 4:**
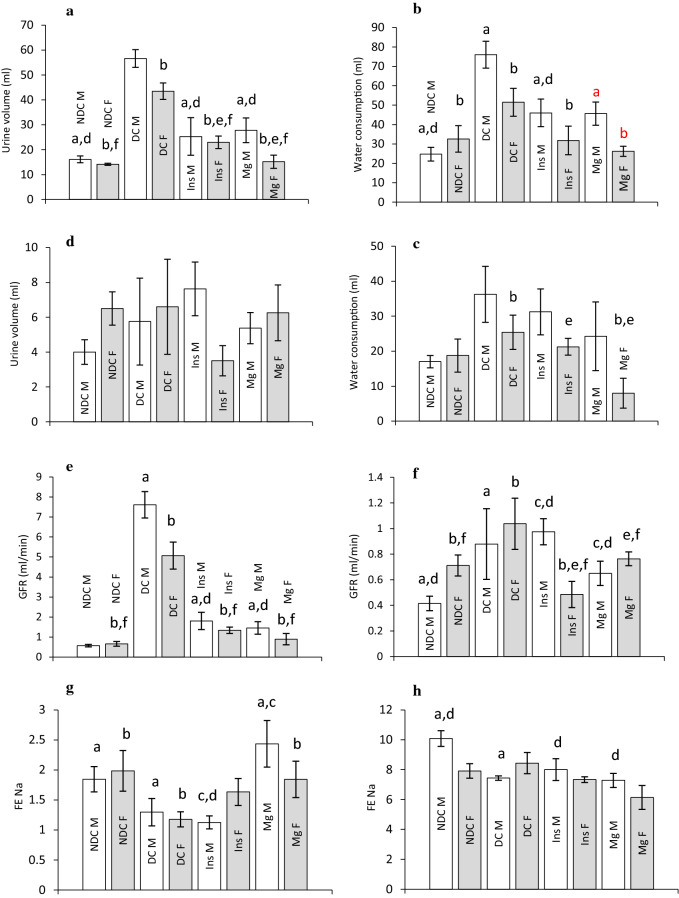

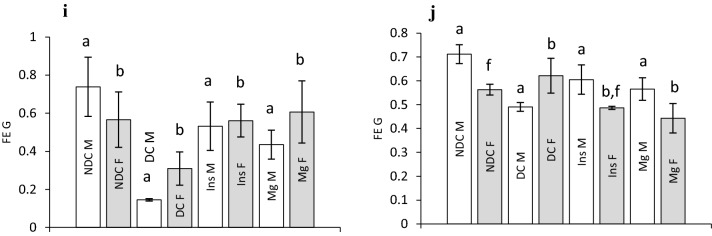
Comparison of water consumption and urine volume in male and female parents and their male and female offspring (**a**, **b**, **c** and **d** respectively), glomerular filtration rate (GFR) in male and female parents and their male and female offspring (**e** and **f** respectively), sodium fraction excretion rate (FE Na) in male and female parents and their male and female offspring (**g** and **h** respectively) and glucose fraction excretion rate (FE G) in male and female parents and their male and female offspring (**i** and **j** respectively) in non-diabetic control group (NDC) was fed with normal diet , diabetic group received high-fat diet and 35 mg/Kg STZ (D), diabetic animals treated with insulin (2.5 U/Kg two time per day) (Ins) and diabetic animals treated with 10 g/l magnesium sulfate via drinking water (Mg). 10 rats in each group and data are expressed as mean ± SEM. (**a**) Significant difference between D male group and other male groups (*P* < 0.0001). (**b**) Significant difference between D female group and other female group (*P* < 0.001). (**c**) Significant difference between male Mg group and male insulin group (*P* < 0.001). (**d**) Significant difference between male NDC and male Mg and male insulin groups (*P* < 0.001). (**e**) Significant difference between female Mg group and female insulin group (*P* < 0.001). (**f**) Significant difference between female NDC and female Mg and female insulin groups (*P* < 0.001).

Water consumption and urine volume were measured in all groups of parents and their offspring (Fig. 4a–d). Our results showed that the urine volume in both parents 3 months after diabetes induction significantly increased (male: *p* < 0.0001, female: *p* < 0.001) in compare to the NDC group (Fig. 4a). Insulin or MgSO_4_ administration could significantly decrease urine volume in compare to DC group in both parents (*p* < 0.001), but it could not reach to NDC group in male treated groups. Water consumption also statistically increased 3 months after diabetes induction in compare to NDC group (male: *p* < 0.0001, female: *p* < 0.001) and insulin or MgSO_4_ therapy could considerably decrease water intake in the both parents, however only in the female Mg-treated and insulin-treated groups the water consumption get to the female NDC group (Fig. 4b). We did not observe any significant differences in water consumption and urine volume between all offspring groups except a significant difference was observed in water consumption in female Mg-treated group with both female DC and female insulin received groups (*p* < 0.001) (Fig. 4c and d).

The results of this study also showed that induction of diabetes in both sexes caused a significant increase (male: *p* < 0.0001, female: *p* < 0.001) in GFR (male 7.6 ± 0.6 ml/min and female 5.07 ± 0.6 ml/min) in compared to both NDC groups (male 0.5 ± 0.05 ml/min and female 0.6 ± 0.1 ml/min) groups (Fig. 4e). MgSO_4_ or insulin administration significantly (*p* < 0.001) decreased GFR in both parents in compared to DC groups (male Mg-treated 1.4 ± 0.3 ml/min, female Mg-treated 0.8 ± 0.2 ml/min, male insulin-treated 1.8 ± 0.4 ml/min and female insulin-treated 1.3 ± 0.1 ml/min).

The results of the present study showed that GFR in both sexes of offspring of DC parents significantly (male: *p* < 0.0001, female: *p* < 0.001) increased in compared to offspring of NDC parents, but only administration of insulin in female offspring could considerably (*p* < 0.001) decrease GFR in compared to female offspring of DC group. No significant changes in GFR were observed in other groups of children (Fig. 4f).

Sodium fraction excretion rate (FE Na) also was measured in both parents (Fig. 4g) and their offspring (Fig. 4h). Diabetes induction significantly (male: *p* < 0.0001, female *p* < 0.001) decreased FE Na in both DC parents in compared to NDC groups. However, insulin therapy only increased FE Na in female parent in compared to diabetic female parent (*p* < 0.001), but Mg therapy increased FE Na in both parents in compared to both diabetic parents (male: *p* < 0.0001, female *p* < 0.001). Our results also showed that FE Na in male offspring of diabetic parents significantly decreased in compared to male offspring of NDC parents (*p* < 0.0001). Insulin or Mg therapy in diabetic parents could not have any effect on FE Na in their offspring. The results of the present study showed that FE G in both parents significantly (mail: *p* < 0.0001, female *p* < 0.001) decreased after diabetes induction and Mg or insulin administration could significantly increase FE G in both sexes (male: *p* < 0.0001, female: *p* < 0.001, Fig. 4i) in compared to DC groups. A significant difference (*p* < 0.0001) was observed in male offspring of DC parents in compared to male offspring of NDC parents. Administration of Mg or insulin significantly (*p* < 0.0001, Fig. 4j) increased FE G in male offspring in compared to DC male children. However, FE G did not change in DC female offspring, but Mg or insulin therapy significantly (*p* < 0.001) decreased FE G in females’ offspring in compared to female’s DC offspring.

**Figure 5:**
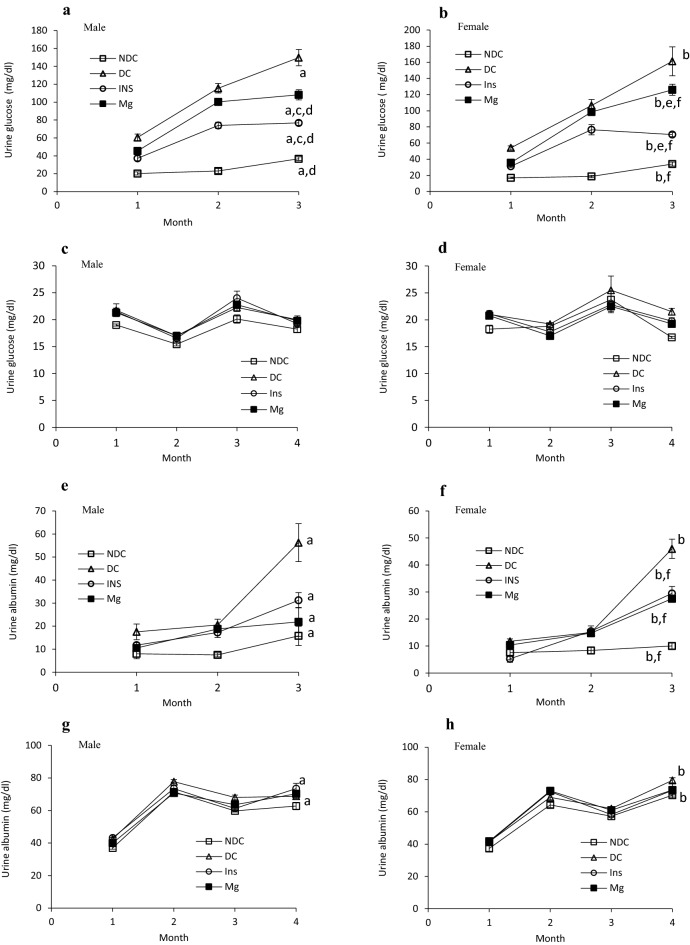

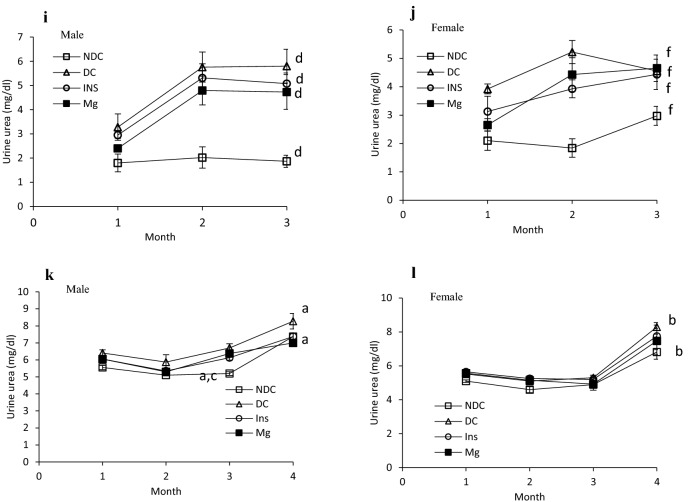
Comparison of urine glucose in male and female parents and their male and female offspring (**a**, **b**, **c** and **d** respectively) and urine albumin in male and female parents and their male and female offspring (**e**, **f**, **g** and **h** respectively) and urine urea in male and female parents and their male and female offspring (**i**, **j**, **k** and **l** respectively) in non-diabetic control group (NDC) was fed with normal diet , diabetic group received high-fat diet and 35 mg/Kg STZ (D), diabetic animals treated with insulin (2.5 U/Kg two time per day) (Ins) and diabetic animals treated with 10 g/l magnesium sulfate via drinking water (Mg). 10 rats in each group and data are expressed as mean ± SEM. (**a**) Significant difference between D male group and other male groups (*P* < 0.0001). (**b**) Significant difference between D female group and other female group (*P* < 0.001). (**c**) Significant difference between male Mg group and male insulin group (*P* < 0.001). (**d**) Significant difference between male NDC and male Mg and male insulin groups (*P* < 0.001). (**e**) Significant difference between female Mg group and female insulin group (*P* < 0.001). (**f**) Significant difference between female NDC and female Mg and female insulin groups (*P* < 0.001).

Urine glucose level was measured in both parents and their offspring in all groups during the study (Fig. 5a–d), our results (Fig. 5a, b) showed that the urine glucose level significantly increased (male: *p* < 0.0001, female: *p* < 0.001) 3 months after diabetes induction in both parents in compared to the NDC male and female groups (female DC group 161.25 ± 17.9 mg/dl, male DC group 149.75 ± 9 mg/dl, female NDC 34 ± 2.3 mg/dl and male NDC group 36.75 ± 2 mg/dl). MgSO_4_ or insulin therapy for 3 months significantly (*p* < 0.001) decreased urinary glucose excretion in both parents (male Mg-treated 108.2 ± 5.7 mg/dl, female Mg-treated 126 ± 6.8, male insulin-treated 76.7 ± 2.5 mg/dl and female insulin-treated 70.5 ± 2.6 mg/dl) in compared to the female and male DC groups, however it could not reach to the NDC groups value (Fig. 5a, b). As Fig. 5a and b show the urinary glucose excretion in both sexes of Mg-treated groups is significantly (*p* < 0.001) more than insulin groups. Our results (Fig. 5c, d) also showed that the amount of urinary glucose excretion in female offspring of DC parents (female 21.5 ± 0.6 mg/dl) was significantly higher (*p* < 0.001) than that of female offspring of NDC parents 4 months after birth (female 16.72 ± 0.1 mg/dl). Female offspring of parents treated with insulin or MgSO_4_ (Fig. 5d) showed a significant decrease (*p* < 0.001) in urinary glucose excretion in compared to female offspring of DC parents 4 months after birth (female offspring of Mg-treated animal 19.25 ± 0.4 mg/dl and female offspring of insulin treated group 19.75 ± 0.6 mg/dl).

The results of the present study showed that urinary albumin level significantly increased (male: *p* < 0.0001, female: *p* < 0.001) 3 months after diabetes induction in both parents (Fig. 5e, f) in compared to both NDC groups (female DC group 46 ± 3.5 mg/dl, male DC group 56.25 ± 8.2 mg/dl, female NDC 10 ± 1 mg/dl and male NDC group 15.7 ± 4.1 mg/dl). Administration of MgSO_4_ or insulin for 3 months significantly decreased (*p* < 0.001) urinary albumin excretion in both parents in compared to both DC groups (male Mg-treated 21.8 ± 6.3 mg/dl, female Mg-treated 27.5 ± 0.8, male insulin-treated 31.25 ± 3.3 mg/dl and female insulin-treated 29.5 ± 2.5 mg/dl). Significant differences were not observed between insulin and MgSO_4_ treated groups in both sexes.

Our data (Fig. 5g, h) also showed that the urinary albumin level excretion in both male and female offspring of DC parents significantly (male: *p* < 0.0001, female: *p* < 0.001) increased in compared to male and female offspring of NDC parents (male DC group 68.7 ± 1.3 mg/dl, female DC group 79.5 ± 1.5 mg/dl, male NDC group 62.7 ± 2.8 mg/dl and female NDC 70.2 ± 0.4 mg/dl). However, administration of MgSO_4_ or insulin in parents could not significantly improved albumin urinary excretion in their offspring 4 months after birth in compared to the offspring of DC parents (male Mg-treated group 70.2 ± 2 mg/dl, female Mg-treated group 73.5 ± 1.1 mg/dl, male insulin-treated group 73.35 ± 4.5 mg/dl and female insulin-treated group 73.5 ± 3.1 mg/dl).

Our findings (Fig. 5i, j) showed that the urinary urea concentration significantly increased (male: *p* < 0.0001, female: *p* < 0.001) in both parents 3 months after diabetes induction in compared to both NDC groups (female DC group 4.5 ± 0.1 mmol/l, male DC group 5.8 ± 0.6 mmol/l, female NDC 2.9 ± 0.3 mmol/l and male NDC group 1.8 ± 0.2 mmol/l). MgSO_4_ or insulin therapy could not decrease urinary urea level excretion in both parents in compared to both DC groups. However urinary urea level significantly (male: *p* < 0.0001, female: *p* < 0.001) increased in both male and female of offspring of DC parents in compared to offspring of NDC parents, but insulin or MgSO_4_ administration could not improve urinary urea level excretion in all of offspring (Fig. 5k and l).

**Figure 6:**
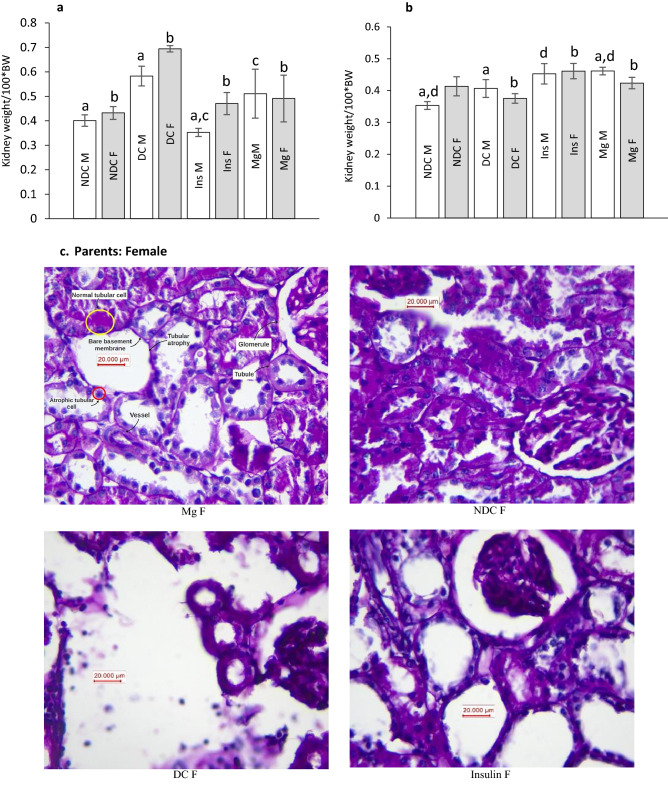

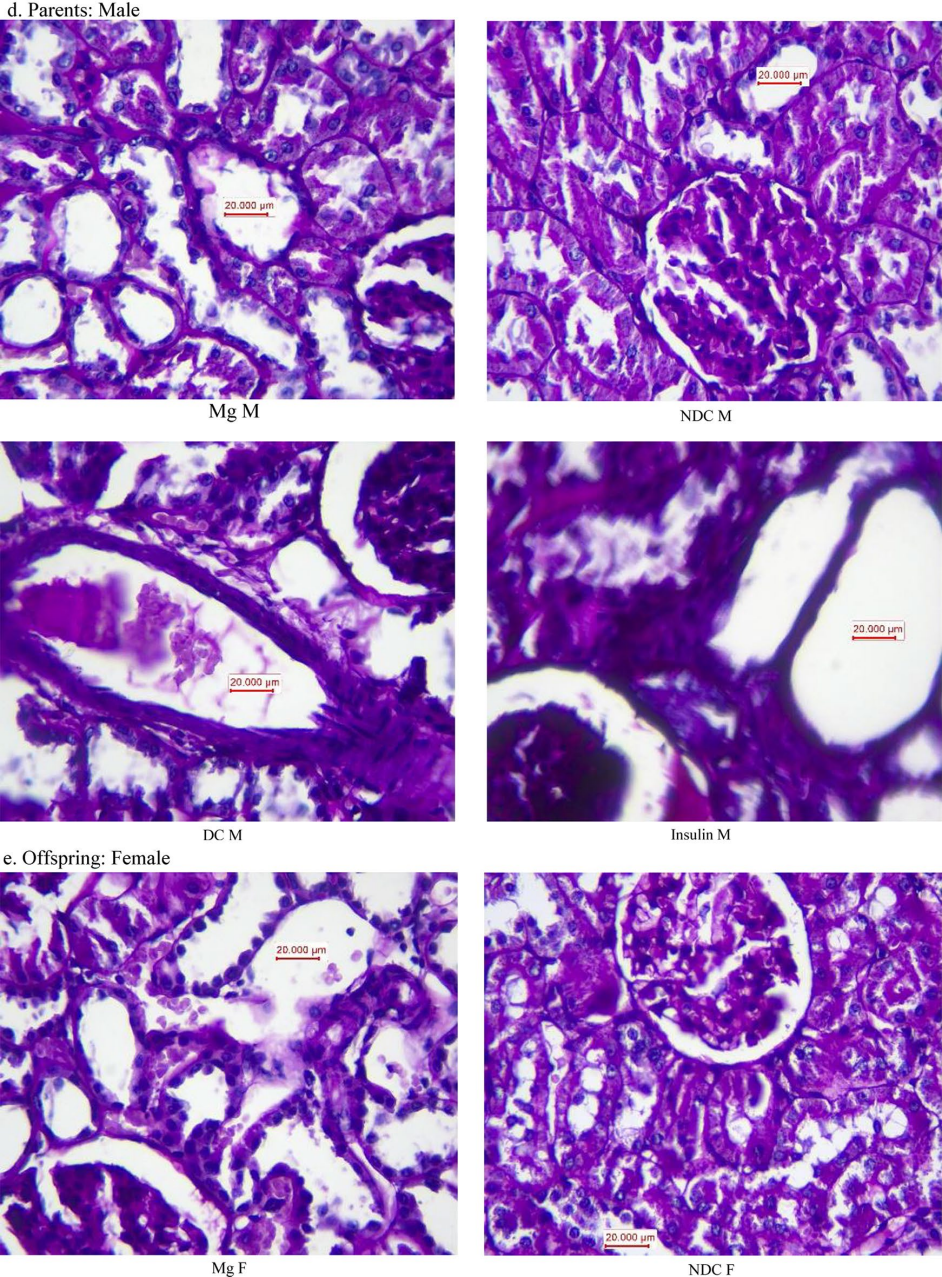

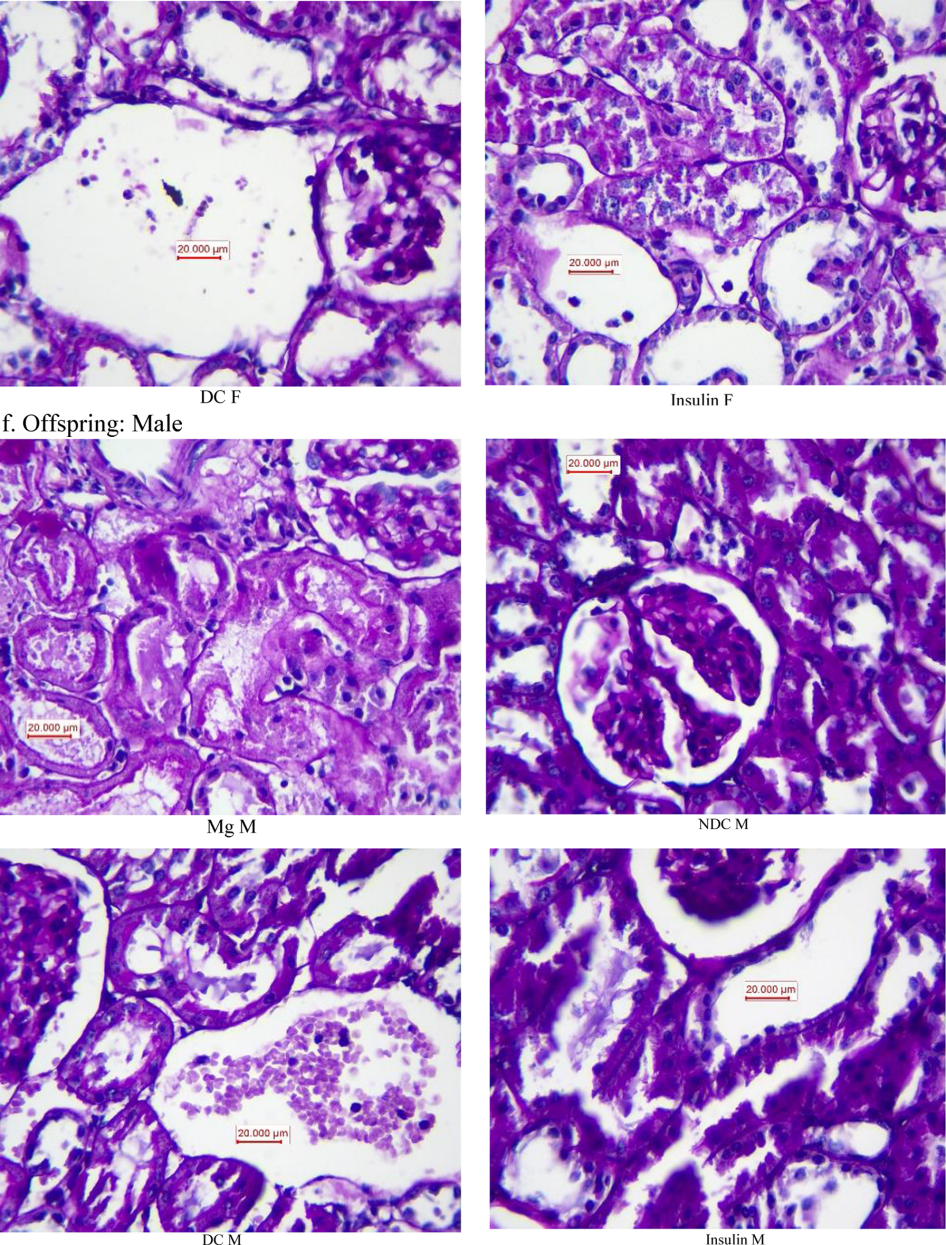
Comparison of kidney weight in male and female parents and their male and female offspring (**a** and **b** respectively) and images of kidney tissues (Magnification × 100) in male and female parents and their male and female offspring (**c**–**f**) in non-diabetic control group (NDC) was fed with normal diet, diabetic group received high-fat diet and 35 mg/Kg STZ (D), diabetic animals treated with insulin (2.5 U/Kg two time per day) (Ins) and diabetic animals treated with 10 g/l magnesium sulfate via drinking water (Mg). 10 rats in each group and data are expressed as mean ± SEM. Kidneys in all groups were weighed using a standard scale by one person immediately after isolation. (**a**) Significant difference between D male group and other male groups (*P* < 0.0001). (**b**) Significant difference between D female group and other female group (*P* < 0.001). (**c**) Significant difference between male Mg group and male insulin group (*P* < 0.001). (**d**) Significant difference between male NDC and male Mg and male insulin groups (*P* < 0.001).

As our results illustrated in Fig. 6a, the kidney weight significantly (male: *p* < 0.0001, female: *p* < 0.001) increased in both parents in DC groups in compared to NDC groups (male DC group 0.58 ± 0.04 g, female DC group 0.4 ± 0.02 g, male NDC 0.69 ± 0.01 g and male NDC 0.43 ± 0.02 g). Insulin therapy in both parents could significantly (*p* < 0.001) decreased kidney weight in compared to both parents in DC groups (male insulin-threated group 0.35 ± 0.01 g and female insulin-treated group 0.47 ± 0.04 g). Administration of MgSO_4_ just could significantly (*p* < 0.0001) decrease the kidney weight in the female animals in compared to female DC animals (female MgSO_4_-treated group 0.49 ± 0.09 g).

Our results which have presented in Fig. 6b shows only the kidney weighs in the male offspring of DC group significantly (*p* < 0.001) increased in compared to male offspring of NDC animals (male NDC group 0.35 ± 0.01 g and male DC group 0.4 ± 0.02 g). And insulin or MgSO_4_ therapy not only could not decrease the kidney weight in compared to both DC groups but also significantly (*p* < 0.001) increased the kidney in compared to both DC groups.

Our pathology results (Fig. 6c–f) showed that renal tubules in both male and female parents of DC groups had been damaged and in some parts of tubules casts were seen in compared to both male and female parents of NDC groups. Tubular size and cellular diameter in both male and female parents of DC groups were more minor than both male and female parents of NDC groups. Bare basements membrane of renal tubules was seen in both male and female parents of DC groups in compared to both male and female parents of NDC groups. Renal tubules cells in both male and female offspring of DC groups have been atrophied and damaged in compared to both male and female offspring of NDC groups and tubular size and cellular diameter in both male and female offspring of DC groups were more minor than both male and female offspring of NDC groups. Bare basements membrane of renal tubules was seen in both male and female offspring of DC groups in compared to both male and female offspring of NDC groups.

No atrophy, damage, tubules casts were seen in renal tubule cells in both male and female parents of insulin or Mg groups and their offspring in compared to both male and female parents of DC groups and their offspring and tubular size and cellular diameter in both male and female parents of insulin or Mg groups and their offspring were bigger in compared to both male and female parents of DC groups. Ordinary basements membrane of renal tubules was seen in both male and female parents of insulin or Mg groups and their offspring in compared to both male and female parents of DC groups and their offspring.

**Figure 7:**
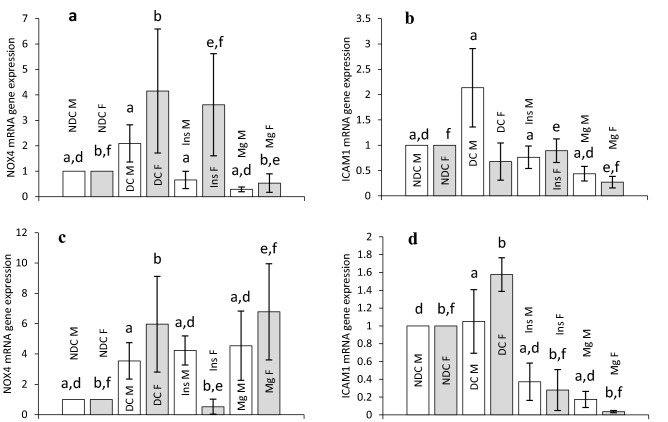
Comparison of NOX4 mRNA gene expression in male and female parents and their male and female offspring (**a** and **c** respectively) and ICAM1 mRNA gene expression in male and female parents and their male and female offspring (**b** and **d** respectively) in non-diabetic control group (NDC) was fed with normal diet, diabetic group received high-fat diet and 35 mg/Kg STZ (D), diabetic animals treated with insulin (2.5 U/Kg two time per day) (Ins) and diabetic animals treated with 10 g/l magnesium sulfate via drinking water (Mg). 10 rats in each group and data are expressed as mean ± SEM. (**a**) Significant difference between D male group and other male groups (*P* < 0.0001). (**b**) Significant difference between D female group and other female group (*P* < 0.001). (**d**) Significant difference between male NDC and male Mg and male insulin groups (*P* < 0.001). (**e**) Significant difference between female Mg group and female insulin group (*P* < 0.001). (**f**) Significant difference between female NDC and female Mg and female insulin groups (*P* < 0.001).

NOX4 mRNA gene expression was measured in all parents and our results were shown in Fig. 7a. As it can be seen diabetes induction significantly (male: *p* < 0.0001, female: *p* < 0.001) increased the NOX4 gene expression in both DC groups in compared to both NDC groups. Administration of MgSO_4_ could significantly (*p* < 0.001) decrease the NOX4 gene expression in both groups in compared to both DC groups, but this action just was seen in the male insulin group in compared to male DC group. Our results also showed that diabetes induction caused a significant increase (male: *p* < 0.0001, female: *p* < 0.001) in ICAM1 gene expression in male group in compared to male NDC animals. Treatment with MgSO_4_ or insulin significantly reduced (*p* < 0.001) the expression of this gene in male groups in compared to male DC group (Fig. 7b).

Figure 7c shows that NOX4 gene expression is significantly increased (male: *p* < 0.0001, female: *p* < 0.001) in both sexes of offspring of DC groups in compared to both NDC parents. And only insulin treatment in female group of offspring was able to reduce (*p* < 0.001) the expression of this gene in compared to female DC group.

The results of ICAM1 gene expression showed that induction of diabetes only in female group of offspring of DC parents markedly (*p* < 0.001) increased the expression of this gene in compared to the female offspring of NDC parents (Fig. 7d). However, insulin or MgSO_4_ administration could significantly reduce (*p* < 0.001) the ICAM1 gene expression in both sexes of Mg-treated or insulin-treated offspring in compared to both male and female DC parents.

The summery of the results are presented in Table 2.

## Discussion

The present study was designed to answer following questions (1) If type 2 diabetes induced by a high-fat diet causes renal dysfunction in male and female rats? (2) Whether or not treatment with insulin or MgSO_4_ can improve kidney function in both male and female diabetic rats? (3) Whether or not impairs renal function seen in offspring of diabetic female and male animals? (4) Is kidney function (GFR, FE Na and FE G) better in offspring of insulin or MgSO_4_-treated parents than in offspring of diabetic female and male animals? The results of this study show that diabetes induction increases IR, urine volume, water consumption, GFR, urine glucose, urine albumin, urine urea, and kidney NOX4 and ICAM1 gene expressions in both female and male mice, as well as in the offspring. Insulin or MgSO_4_ administration improves FE Na, FE G, IR, urine volume, water consumption, GFR, urine glucose level, urine albumin level, urine urea and kidney NOX4 and ICAM1 gene expressions in female and male animals and their offspring.

IPGTT, GIR, and blood glucose levels are considered an IR index. This study's findings showed that diabetes induction increases IR and blood glucose levels in both male and female animals compared to both NDC groups. MgSO_4_ therapy significantly decreased IR and blood glucose levels in male and female groups compared to DC groups. Our previous study^26,27^ showed that MgSO_4_ administration could decrease IR and blood glucose in diabetic animals, that part of these actions are mediated by muscles, because in our previous work^26,27^ we showed that MgSO_4_ therapy in T2D rats could increase GLUT4 gene expression and translocation on the cell membrane. We showed that MgSO_4_ treatment improved kidney function and decreased blood glucose levels. Because of the finding that MgSO_4_ treatment increases urine glucose excretion in comparison to both NDC groups in both parents. Researchers reported some medication like empagliflozin improves T2D and reduces blood glucose levels by inhibiting selective sodium glucose co‐transporter 2 and increasing urinary glucose excretion^35^. DeFronzo et al. showed that renal glucose reabsorption increases in T2D patients^36^. So, the suppression of renal glucose reabsorption is a powerful method to treat diabetes. On the other hand, our results showed that the urinary glucose excretion in the offspring of MgSO_4_-treated groups increased compared to the offspring of both NDC groups, but blood glucose level were the same which has a normal range in all groups of offspring. GIR increased in both sexes of MgSO_4_ or insulin-treated offspring compared to the offspring of DC groups. However, the increase in urinary glucose excretion was observed in insulin-treated parents compared to both NDC groups, and blood glucose level decreased in these groups compared to DC groups. But, GIR the golden standard index for IR is still low in both insulin groups compared to male and female of DC groups. So, it seems to increase the excretion of glucose in the urine alone, although it can reduce blood sugar, it cannot improve IR. Large kidneys are a factor to predict the development of advanced chronic kidney disease, and renal hypertrophy develops in diabetic nephropathy (CKD)^37^. Our findings showed that, as compared to the NDC groups, kidney weight rises with T2D induction in both sexes. Insulin or MgSO_4_ therapy could significantly decrease kidney weight in both parents compared to D groups. GFR and inflammation have a negative relationship. Researchers have shown that kidney inflammation may worsen IR because it generates a large amount of pro-inflammatory bioactive proteins that itself can induce IR^38^. Kidney inflammation was not observed in the offspring of DC groups compared to the offspring of NDC group, however insulin or MgSO_4_ therapy slightly increased KW, but the inflammation was not enough to increase IR. Mg supplementation reduced inflammation associated with CKD and the other study showed that dietary Mg supplementation reduced the systemic inflammation^39^.

Hypomagnesemia impairs kidney function in renal transplant recipients and diabetics^40^. Kanbay et al., in their cohort study, reported that Mg deficiency contributes to cardiovascular outcomes and plays an important role in etiologically diagnosed CKD patients^41^. Tin et al. reported that low serum Mg levels are associated with kidney function in the non-diabetic population^42^. According to a research, the kidney's filtration-reabsorption mechanism largely controls the Mg homeostasis. In contrast to insulin, low plasma Mg levels reduced Mg reabsorption from the distal tubule^43^. We showed that the serum Mg level fell following T2D induction in both this study and our earlier one^27^. Low level of Mg in the was recently shown to be a novel risk factor for incident CKD and incident end-stage renal disease^44^. Hypomagnesemia, in turn, increases IR in T2D. On the other hand, IR is a common risk factor to develop cardiovascular disease and CKD. Decreased response to insulin is already manifested in mild renal dysfunction and progresses with declining GFR^45^. It is not identified if IR by itself contributes to increase risk of both CKD progression or CKD complications^46^. We demonstrated that IR might harm kidney health. According to our research, both male and female diabetic parents' GFR considerably increased as compared to both NDC groups. Because IR causes hyperglycemia and blood glucose levels to rise, which in turn causes the filtration rate to rise and, as a result, the GFR. An extended increase in GFR might lead to end-stage renal disease in the patient^47^. As our results showed that urine albumin excretion significantly increased in both diabetic parents compared to both NDC groups. Albuminuria was observed in both DC groups compared to both NDC groups. However, MgSO_4_ or insulin therapy could decrease GFR and urine albumin excretion in both sexes compared to both DC groups, but they did not decrease urine urea excretion in both sexes compared to both NDC groups. On the other hand, increased GFR following induction of diabetes in both parents reduces FE Na and FE G compared to the NDC groups. In comparison to both NDC groups, insulin or MgSO_4_ medication in both parents lowers GFR, which in turn lowers both parents' FE Na and FE G levels. The same pattern was observed in male children of diabetic parents and parents receiving MgSO_4_ or insulin, Ferrannini et al. showed that acute insulin infusion stimulates renal glucose and sodium excretion, and this effect may be mediated by sodium glucose transporter (SGLTs)^48^. FE Na can be useful to evaluate acute kidney failure. Low FE Na indicates sodium retention by the kidney, and higher values of FE Na can suggest sodium wasting in terms of the acute tubular necrosis or other causes of intrinsic kidney failure^49^. Our results showed that urine volume and water consumption significantly increased in both DC parents compared to NDC parents. Furthermore, insulin or MgSO_4_ therapy significantly decreased urine volume and water consumption in both parents compared to DC groups. Our previous studies showed that Mg or inulin administration could decrease some signs of diabetes, such as urine volume and water intake, because they can decrease blood glucose levels^26,27^. Blood glucose increases renal tubular osmolality which in turn does not allow the water to reabsorb in the kidney. The lack of water reabsorption from the kidneys increases plasma osmolality and stimulates the thirst center in the brain.

The results of the present study showed that T2D induction, increases NOX4 gene expression in both parents compared to NDC parents. While insulin treatment could only reduce NOX4 gene expression in the male group, MgSO4 therapy could do the same for both sexes. NOX4 is a major source of renal reactive oxygen species (ROS) and oxidative stress, and it produces on epithelial and mesangial cells^50^. We speculate that anti-inflammatory effect of Mg and normalizing hyperfiltration by MgSO_4_ or insulin administration may have contributed to the suppression of NOX4 production. Yang et al. reported that Mg supplement blocked NOX1, NOX2 and NOX4 gene expressions^51^. NADPH-oxidase activity and its function are low in cerebral arteries of female rats. However, Miller et al. showed that NOX1, and NOX4 gene expressions are estrogen-dependent, but insulin could not decrease NOX4 gene expression in the female animals^52^. High fat diet increases NOX4 gene expression, and it in turn is efficient to trigger IR^53^, so it seems that a part of IR in female insulin group is mediated by oxidative stress. Despite researchers’ clams about the beneficial effects of insulin in renal disease^54^, but due to the insulin is involved in the reabsorption of glucose and sodium from the proximal tubule as well as up taking of glucose into the podocyte cells in the glomerular membrane, insulin has different effects on the kidneys related to blood glucose and urine glucose levels and the severity of IR. Only MgSO_4_ treatment was able to dramatically reduce NOX4 gene expression in the female progeny of DC animals, since NOX4 gene expression rises in the offspring of DC animals. ICAM1 is a primary candidate to develop DN via increased vascular inflammation and fibrosis. High blood glucose level increases endothelial ICAM1 expression, and an individual study showed a direct relationship between renal ICAM1 expression and progressive renal injury in animal models of DN^55^. Our results showed that diabetes induction just increases ICAM1 gene expression in the male group compared to male NDC group. Another study found that ICAM1 in a male rat model of diabetes plays a key role to promote the macrophage infiltration in glomeruli and they showed that block the activity of ICAM1 ameliorates DN^50^. Our results showed that, as compared to the female NDC group, diabetes induction had no effect on the expression of the ICAM1 gene in the female group. Honjo et al. provided evidence that estrogen reduced ICAM1 expression^56^. Our results showed that insulin or MgSO_4_ therapy could significantly decrease ICAM1gene expression in both parents compared to both NDC parents. Victor et al. showed that the usage of insulin plays an important role to reduce the level of ICAM1 in diabetic patients^57^. Mg plays physiological roles in cardiovascular function. Mg deficiency affects cardiovascular health, endothelial cell function, and endothelial cell proliferation, increasing monocyte adhesion and markedly altering endothelial cell gene expression^58^. Orhan et al. reported that Mg supplementation significantly decrease the levels of NF-κB, INOS, ICAM in HFD rats^59^. Our findings demonstrated that the female offspring of the DC group had considerably higher ICAM1 mRNA gene expression as compared to the female NDC group. However, as compared to the male and female DC groups, ICAM1 mRNA gene expression was dramatically reduced by either MgSO4 or insulin treatment in both male and female animals.

## Conclusion

This study's results indicated that type 2 diabetes induction decreased serum Mg level, and hypomagnesemia in turn increased IR in type 2 diabetes. On the other hand, insulin resistance is a common risk factor to develop cardiovascular disease and chronic kidney disease (CKD). MgSO_4_ therapy significantly decreased some signs of diabetes, such as IR and blood glucose levels in male and female rats and their offspring. However, we demonstrated that MgSO_4_ treatment may enhance renal function and lower IR in diabetic animals. While it could seem that increasing urine glucose excretion might lower blood glucose levels, this cannot be used to reduce insulin resistance. Maybe MgSO_4_ plays an important role in DN via decreasing IR. Regarding the limitation to provide radioactive glucose, we could not calculate the exact contribution of the kidney to the total insulin resistance. Therefore, it seems difficult to understand whether the kidney is a target cell of insulin or not.

## Data Availability

The datasets used and/or analysed during the current study available from the corresponding author on reasonable request.
